# Ocular Effects of MEK Inhibitor Therapy: Literature Review, Clinical Presentation, and Best Practices for Mitigation

**DOI:** 10.1093/oncolo/oyae014

**Published:** 2024-03-25

**Authors:** Karen W Jeng-Miller, Miles A Miller, Jeffrey S Heier

**Affiliations:** Ophthalmic Consultants of Boston, Boston, MA, USA; Center for Systems Biology, Massachusetts General Hospital Research Institute, Boston, MA, USA; Department of Radiology, Massachusetts General Hospital and Harvard Medical School, Boston, MA, USA; Ophthalmic Consultants of Boston, Boston, MA, USA

**Keywords:** MEK inhibitor, retinopathy, MEKAR, ocular adverse events

## Abstract

MEK signaling pathway targeting has emerged as a valuable addition to the options available for the treatment of advanced cancers including melanoma and non-small cell lung cancer. Ophthalmologic monitoring of patients taking part in clinical trials of MEK inhibitors has shown that while ocular effects are common, generally emerging during the first days to weeks of treatment, the majority are either asymptomatic or have minimal visual impact and are benign, resolving without intervention or the need to reduce or stop MEK inhibitor therapy. However rare cases of serious, potentially vision-threatening ocular toxicities have been reported during MEK inhibitor therapy. There is currently no recommendation for routine ophthalmologic screening or monitoring of patients with advanced cancer who are initiating MEK inhibitor therapy. However, baseline ophthalmologic examination may be useful for all patients initiating MEK inhibitor therapy to allow the differentiation of preexisting pathology versus the development of MEK inhibitor-associated retinopathy in the event of the emergence of symptomatic ocular events. Regular ophthalmologic examination may be appropriate for patients at increased risk for ocular events, such as patients with a history of ocular inflammation, infection, or underlying macular/retinal disease. All patients reporting visual disturbance should be referred for prompt ophthalmologic review to determine the potential seriousness of any underlying abnormalities and whether there is a need for treatment modification or specific intervention. Understanding the potential consequences of ocular toxicities is of particular importance in the context of decision-making for the continuation of potentially life-prolonging medications such as MEK inhibitors.

## Teaser

Serious ocular adverse events have emerged as a rare but important consequence of MEK inhibitor therapy, thus requiring early ophthalmic screening. This article reviews the ocular adverse events associated with MEK inhibitor therapy for advanced cancers, with a particular focus on MEK inhibitor-associated retinopathy, and reports clinical experience and best practice approaches to management.

**Implications for Practice**

Ocular toxicities are a known class effect of MEK inhibitor therapy. The majority of symptomatic ocular toxicities are likely to resolve without clinical sequelae or the need for the interruption or cessation of potentially life-prolonging MEK inhibitor therapy. However, the potential for vision-threatening ocular toxicities associated with MEK inhibitor therapy, while small, suggests a need for ophthalmologic examination before treatment is initiated. Examination of patients at risk for such events, including those with a history of ocular inflammation, infection, or underlying macular/retinal disease, should be routine practice with MEK inhibitor therapy.

## Introduction

The mitogen-activated protein kinase (MAPK) pathway plays a critical role in cellular signaling and contributes to the regulation of a variety of processes including cell proliferation, survival, differentiation, motility, and angiogenesis.^1,2^ The fundamental components of the pathway consist of the Ras small GTPases, which recruit and activate the downstream kinases RAF and MEK (MAP kinases) that in turn trigger the activation of the extracellular signal-regulated kinase (ERK) proteins.^3^ Activated ERKs can either translocate to the cell nucleus, with implications for gene expression, or remain in the cell cytoplasm where they interact with a variety of substrates.^1,3^ The subcellular location of ERK proteins can determine their role in regulating diverse cell functions.^4,5^ Through variations in the individual components, this basic pathway structure enables cells to detect extracellular signals and respond by driving or inhibiting diverse cellular functions.

Aberrant MAPK signaling is implicated in the development and progression of >40% of all human cancers, including melanoma, colorectal, non-small cell lung, thyroid, pancreatic, breast, and endometrial cancers.^2,3,6^ For this reason, the MAPK pathway has been subject to intense investigation in order to better understand the pathophysiologic basis of human carcinogenesis as well as for the development of novel therapies.

Agents targeting the MEK components of the MAPK signaling cascade have been investigated for the treatment of various tumor types in recent years, with promising results.^1^ Seven MEK enzymes (MEK1-7) have been identified so far that operate within 4 distinct MAPK signaling pathways and selectively activate downstream substrates of the signaling cascade.^1^ Inhibitors targeting MEK1 and MEK2 have received the most attention, with several, including trametinib, cobimetinib, binimetinib, and selumetinib,^7-10^ approved for clinical use. Trametinib is approved for the treatment of unresectable/metastatic melanoma (with a *BRAF* V600 mutation), advanced non-small cell lung cancer (with a *BRAF* V600 mutation), and advanced anaplastic thyroid cancer (with a *BRAF* V600 mutation), all in combination with the BRAF-targeted kinase inhibitor dabrafenib. The MEK1/2-targeted inhibitors cobimetinib (in combination with the BRAF-targeted vemurafenib) and binimetinib (in combination with the BRAF-targeted encorafenib) are approved for the treatment of adults with *BRAF* V600-mutant unresectable or metastatic melanoma, and the MEK1/2-targeted inhibitor selumetinib is approved for the treatment of pediatric neurofibromatosis type 1. These agents have been shown to decrease tumor burden and prolong progression-free and overall survival.^11-15^ However, as with most targeted therapies, MEK inhibitors are associated with adverse events. For this class, the predominant adverse events include papulopustular exanthema, serous retinal detachments, musculoskeletal events, hypertension, and decreased ventricular ejection fraction.^16^ While most such events are mild or moderate in intensity and time-limited, vigilance for their emergence and appropriate interventions to ameliorate their effects is an important part of the management of patients receiving targeted therapy.

Serious ocular adverse events have emerged as a rare but important consequence of MEK inhibitor therapy, thus requiring early ophthalmic screening and subsequent surveillance.^17,18^ Here we review the ocular adverse events associated with MEK inhibitor therapy for advanced cancers, with a particular focus on MEK inhibitor-associated retinopathy (MEKAR), and share clinical experience and best practice approaches to their management.

## Ocular Adverse Events Associated With MEK Inhibitors

Ocular adverse effects related to MEK inhibitors are mostly concentrated in the retina. These effects include clinical findings of chorioretinopathy and serous retinal detachment, which, if symptomatic, present with symptoms of blurred vision, halos around lights, and colorful spots in the vision.^11,17,19-21^ Although many patients with ocular adverse effects are asymptomatic or have only mild symptoms, some of these effects can have vision-threatening potential.^17^ Therefore, these side effects are important for practitioners to be aware of as MEK inhibitor usage increases.

MEKAR is characterized by the presence of subretinal fluid resulting in the self-limiting serous detachment of the neurosensory retina.^19,22,23^ The pathophysiologic basis of MEKAR is, as yet, not completely understood, but appears to be caused by acute toxic effects on the retinal pigment epithelium and resultant dysfunction by inhibition of the MAPK pathway that lies downstream of the fibroblast growth factor receptor (FGFR).^24^ The FGFR binds a neurotrophic factor that is essential for maintenance, repair, and survival of the retinal pigment epithelium. MEK inhibition causes increased permeability of the retinal pigment epithelium and subsequent accumulation of subretinal fluid via upregulation of aquaporin 1, a membrane water channel essential in the permeability of the retinal pigment epithelium.^25,26^

Indirect evidence suggests local disruption of the blood-retinal barrier may also be involved in MEKAR. In mice, genetic Erk1/2 ablation systemically disrupts the integrity of quiescent endothelium; in rats, treatment with the MEK inhibitor PD0325901 causes evidence of retinal endothelial damage; and in rabbits, PD0325901 treatment can elicit retinal vein occlusion.^27,28^ Microglia are tissue-resident macrophages of the retina that maintain physiologic blood-retinal barrier integrity, and trametinib can induce macrophages to produce factors, including vascular endothelial growth factor (VEGF), that signal locally with neighboring cells.^29^ Despite possible roles for perivascular signaling, MEKAR does not present with neovascularization expected from chronic VEGF-driven angiogenesis.

MEKAR has been estimated to occur in up to 90% of patients receiving MEK inhibitor therapy, although the majority of cases are apparently asymptomatic and resolve without the need for intervention or compromising the therapeutic dose of cancer-directed therapy.^19,23,30^ Clinical trial data show a high incidence of MEKAR among treated patients but also indicate that only a minority of patients have symptomatic MEKAR. For most of these patients, the events are self-limiting and resolve without the need for intervention or dose adjustment.^14,18,31,32^

## Screening for Ocular Toxicity During MEK Inhibitor Therapy

There is currently no recommendation for the routine ocular screening of patients undergoing MEK inhibitor therapy. Previous MEK inhibitor studies recommended ocular screening of patients based on incidences of ocular adverse events observed; some ocular adverse events, such as conjunctivitis, were detected as part of routine physical examination of patients, while others, including retinopathy, required ophthalmologic examination.^17^ A baseline ophthalmologic evaluation, including visual acuity and fundoscopy, may be useful for all patients initiating MEK inhibitor therapy.^17^ A baseline exam will allow differentiation of preexisting pathology from MEKAR. This distinction may be important in the case of symptomatic pathology and may impact treatment. For instance, if symptomatic disease is due to MEK therapy, dose modification or hold may be necessary. However, if symptoms are due to underlying disease progression, for instance, worsening of an epiretinal membrane, there is no need or benefit to changing MEK therapy. Regular screening should be considered for patients who may be at increased risk for ocular toxicities, such as those with underlying macular disease which could make recognition of ocular toxicities difficult. For patients reporting visual disturbance, prompt ophthalmology referral is appropriate. A baseline ophthalmologic examination, including visual acuity, slit-lamp and dilated funduscopic examination, and optical coherence tomography imaging, establishes a baseline and may uncover any underlying conditions that could confound the detection of MEKAR and its subsequent management.

## Report of Cases With Ocular Toxicity

### Presentation 1: Mild Asymptomatic

*Describe/discuss:* Asymptomatic ocular toxicities associated with MEK inhibitor therapy have been detected among patients participating in clinical trials who have undergone regular ophthalmologic examination. Visual acuity changes may be apparent on examination even when vision changes are not reported by the patient (Table 1). Retinal lesions may be observable on examination and optical coherence tomography may reveal changes such as subretinal fluid both in subfoveal and extrafoveal locations. In the case of a patient receiving MEK inhibitor therapy for metastatic ovarian cancer, such lesions were apparent on optical coherence tomography 2 weeks after initiation of MEK inhibitor therapy.^33^ In our experience, subretinal fluid can be present even as early as a few hours after initiation of MEK inhibitor therapy. In this case, treatment was continued because the changes were not considered to be vision-threatening, and the lesions had almost completely resolved by 4 weeks after MEK inhibitor therapy initiation without the need for dose reduction or treatment interruption. In instances similar to this presentation, observation is recommended. It is not recommended that the systemic treatment be discontinued. Repeat ocular examination in 4 weeks is appropriate, sooner if any vision changes should arise.

**Table 1.** Clinical presentations observed with ocular toxicity.

	Grade	Symptoms	Management
Mild asymptomatic	1	None	Monitor. Follow-up in 4 weeks for re-examination
Mild symptomatic	2	Mild transient or persistent blurred vision that does not impact activities of daily living	Monitor. Follow-up examinations in 2-4 weeks. Continue systemic medication
Moderate	3	Moderate persistent blurred vision that does impact activities of daily living	Consider dose de-escalation or cessation. Medication re-initiation can be trialled after retinopathy resolves
Severe	4	Persistent blurred vision with severe impact on activities of daily living	Recommend trial dose de-escalation or cessation. Medication re-initiation can be trialled after retinopathy resolves

### Presentation 2: Mild Symptomatic

*Describe/discuss:* Mild or transient visual symptoms, such as blurred vision, reported by patients may indicate ocular toxicities and warrant ophthalmologic investigation (Table 1). The emergence of blurred vision may be associated with serous retinal detachment (detectable on optical coherence tomography) as well as uveitis.^34^ Such was the case for 2 patients receiving MEK inhibitor therapy for metastatic cancer.^34^ For both patients, neurosensory subfoveal retinal detachment was detected on optical coherence tomography. For both patients, the abnormality resolved after 1 week; 1 patient required no change to MEK inhibitor dosage and the other patient required a dose reduction. The decision to decrease the dose or dose hold is largely based upon the degree of the patient’s symptoms. If mild, and not impacting daily functioning, continuing the current dose is reasonable, but with closer follow-up to ensure visual stability. Repeat ophthalmologic examination in 2-4 weeks, or sooner if symptoms increase, is appropriate.

### Presentation 3: Moderate With Visual Symptoms

*Describe/discuss:* Patient-reported blurred vision may indicate more concerning underlying ocular effects during MEK inhibitor therapy including macular edema requiring additional intervention^33^ (Table 1). The case reported by Duncan et al highlights the potential for such events to occur later during the course of MEK inhibitor therapy. In this case, the patient reported the emergence of blurred vision 8 months after the initiation of MEK inhibitor therapy. Retinal examination and optical coherence tomography revealed cystoid macular edema of the left eye. The patient had no history of diabetes, uveitis, macular degeneration, eye surgery, vein occlusions, or any other etiology for the disease. The patient’s macular edema was treated with prednisolone acetate 1% (Pred Forte) and ketorolac (Acular) topical drops with complete resolution after 6 weeks. Ongoing treatment with anti-inflammatory eye drops was required to prevent recurrence; however, this is uncommon with MEKAR and it may have been secondary to an unrecognized, unrelated etiology. It should be noted that cystoid macular edema is an uncommon occurrence with MEK inhibitor therapy and may have been secondary to an unrecognized, unrelated etiology.^33^ However, in the case of moderate symptoms impacting the patients’ daily activities, dose reduction or dose holding is common. The retinopathy often resolves quickly, typically within 1 to several weeks, allowing resumption of MEK therapy. If the dose was held, then resumption at a lower dose is frequently used. If the dose was reduced, observation for further retinopathy, and retinopathy does not recur, increasing the dose slowly may be tried.

### Presentation 4: Severe With Visual Symptoms

*Describe/discuss:* More severe ocular toxicities may also emerge during MEK inhibitor therapy and can be associated with vision loss and pain. Such was the case for a patient receiving MEK inhibitor therapy for metastatic melanoma who experienced vision loss, pain, and foreign body sensation in both eyes 3 months after the initiation of MEK inhibitor therapy^35^ (Table 1). The patient reported no prior medication, relevant illnesses, or allergies. A uveitis work-up, including blood test and computer tomography, was negative. Serous retinal detachment of the fovea was revealed on fundoscopy and optical coherence tomography. MEK inhibitor therapy was stopped immediately, although anterior uveitis developed 2 weeks later requiring treatment with topical corticosteroids and subsequently periocular triamcinolone acetonide injection.^35^ In our opinion, it is unlikely that the uveitis was associated with MEK inhibitor therapy. Association with ocular inflammation has been documented, albeit with a limited sample size, in the phase III coBRIM study (4/5 patients with a history of ocular inflammation developed symptomatic retinopathy among 247 patients treated with cobimetinib + vemurafenib).^31^ For this patient, MEK inhibitor therapy was initiated again after 6 months, and although serous retinal detachment was detected again, no inflammatory process or uveitis emerged.^35^ With severe ocular symptoms, it is reasonable to trial cessation of the medicine to observe if there is a swift resolution of the symptoms. Like in cases of moderate toxicity, the retinopathy often resolves swiftly allowing for re-introduction of the MEK inhibitor therapy. Close follow-up is advised and dose escalation trials may be attempted over time.

## Discussion

Ocular toxicities are listed as adverse effects for all currently approved MEK inhibitors and monitoring requirements and recommendations for discontinuation of treatment for non-resolution of symptomatic events are included for cobimetinib, trametinib, binimetinib, and selumetinib.^7-10^ Ophthalmologic evaluations are recommended for all patients, including those receiving binimetinib, who report visual disturbance.^7,8,10^ While the incidence of retinal vein occlusion is low among patients receiving MEK inhibitor therapy, retinal vein occlusion is a serious adverse event and, in such cases, systemic evaluation to rule out other risk factors may be considered.^36^ Prescribing information dictates to permanently discontinue MEK inhibitor therapy if retinal vein occlusion is confirmed.^7,36^ Retinal vein occlusion is the second most common retinal vascular disease in the US behind diabetic retinopathy, and patients with cancer are at higher risk given their hypercoagulable state.^37,38^ In our clinical experience, we have rarely, if ever, treated retinal vein occlusion in our MEK inhibitor patients that could be directly attributed to MEK inhibitor therapy. Based on clinical trial data, the prevalence of retinal vein occlusion in patients receiving MEK inhibitor therapy is 0.5%, which is higher than the 0.1% prevalence in the general population.^36^ The increased incidence could be related to MEK inhibitor therapy, but it could also be secondary to confounding factors such as hyperviscosity related to their underlying disease. Retinal pigment epithelial detachment is more frequently observed, and regular ophthalmologic examinations upon initiation of MEK inhibitor therapy are warranted.^7,8,10^

Registrational phase II and prospective observational studies conducted for cobimetinib and binimetinib provided valuable insights into the prevalence, course, and long-term sequelae of MEKAR in patients with advanced cancers. In the phase III coBRIM study among patients with *BRAF-*mutated melanoma receiving treatment with cobimetinib + vemurafenib (*n* = 247) or vemurafenib alone (*n* = 246), 86 cases of serous subretinal fluid were reported among 69 patients, 62 in the cobimetinib group and 7 in the vemurafenib group.^18^ Despite this apparently high incidence of serous retinopathy among patients treated with cobimetinib, cases were generally asymptomatic or mild and resolved without the need for dose modification or other intervention.^18^ More recent data from the coBRIM study have provided additional insights into the ocular adverse events associated with this agent.^31^ Among the total cohort, 72 patients (29%) developed retinopathy. Although most cases were asymptomatic, 33 patients (13%) developed symptomatic retinopathy. The most prominent risk factor for retinopathy was a history of ocular disease, including ocular inflammation. For patients with a history of ocular inflammation, infection, or significant irritation of the eye, 4 out of 5 developed symptomatic retinopathy during treatment.^31^

MEKAR has also been reported in clinical trials among patients treated with binimetinib.^14,32^ These events appear early, often within the first few hours or days of treatment, and are usually mild and transient, resolving without the need for intervention or dose modification. In the phase III COLUMBUS study, in which patients with *BRAF*-mutant melanoma received encorafenib + binimetinib (*n* = 192), encorafenib alone (*n* = 192), or vemurafenib alone (*n* = 186), all patients were required to undergo regular ophthalmologic examination due to signals from previous MEK inhibitor studies, and patients were closely monitored for serous retinopathy in those receiving binimetinib via frequent routine eye assessments.^14,32^ The incidence of serous retinopathy in the combination arm of the study was 20%, with the majority (87% of all events in this group) being grade 1 or grade 2.^32^ Dose interruption or adjustment was required for 11 patients, with no patient requiring discontinuation of the study drug for the resolution of this event. The majority of events were classed as recovered (82%) or recovering (3%), suggesting a general reversibility of serous retinopathy when associated with binimetinib treatment.^32^

Beyond the registrational studies for binimetinib, the emergence of serous retinopathy was evaluated in a prospective, observational, cohort-based, cross-sectional study of 30 patients with cutaneous melanoma and 5 patients with uveal melanoma treated with binimetinib or a combination of binimetinib and the protein kinase C inhibitor sotrastaurin.^21^ Optical coherence tomography revealed accumulation of subretinal fluid (indicating MEKAR) among 77% of patients with cutaneous melanoma and 60% of those with uveal melanoma, affecting the fovea in 85% of cases.^21^ Six patients were evaluated for the presence of autoantibodies against retinal pigment epithelium proteins, and their presence was confirmed in all 6 patients, suggesting that autoantibody formation in addition to drug-related toxicity may play a role in binimetinib-associated serous retinopathy.^21^ In a separate prospective, observational study in a cohort of 32 patients with advanced cutaneous melanoma, treatment with binimetinib as a single agent or in combination with RAF inhibitors induced transient retinopathy with multiple bilateral lesions in some patients.^30^ Patients underwent regular ophthalmologic examinations that revealed grade 1-2 retinopathies in 13/20 patients receiving binimetinib monotherapy and 6/12 patients receiving combination therapy. Patients reported mild and short-lived visual disturbances and the increase in central retinal thickness observed during the initial weeks of treatment markedly decreased during continued treatment.^30^ A longer-term (up to 2 years) evaluation of MEKAR in patients with metastatic melanoma receiving treatment with binimetinib found that although almost all patients showed signs of MEKAR during the initial weeks of treatment, the retinopathy resolved over time without any reported functional deficits in vision or changes in the structural integrity of the retina.^30^

Routine ophthalmologic evaluations of patients taking part in the registrational trials for trametinib in melanoma and non-small cell lung cancer were not conducted, so the incidence of MEKAR in trametinib-treated patients is unknown.^7^ There is limited information on ocular adverse events during treatment with selumetinib. In the phase II registrational SPRINT study that evaluated selumetinib in 74 patients in pediatric care with neurofibromatosis type 1, ocular adverse events were reported in 15% of patients and resolved in 82% of cases.^10^

Routine ophthalmologic examination is not required for patients initiating MEK inhibitor therapy, but it can be helpful in differentiating underlying pretreatment retinal pathology from MEKAR, and subsequently, impacting the type of treatment used to improve vision. However, the potential for ocular side effects, for which the outcome and course are still unknown, suggests that an initial ophthalmologic examination is warranted to enable clinicians to recognize MEKAR from underlying disease. This would allow for clinically directed recommendations regarding dose modification, treatment, and additional regular ophthalmologic monitoring. Further understanding of ocular effects associated with MEK inhibitor therapy is needed to better understand the evolution and appropriate management of these adverse events, identify patients most at risk, and prevent unnecessary interruption of therapy for asymptomatic or self-limiting MEKAR.

A current challenge in the evaluation of the true prevalence of potentially vision-threatening ocular events relates to the variability of ocular toxicity report descriptions. For example, in clinical trials, the term serous retinopathy may encompass retinal detachment, chorioretinitis, chorioretinopathy, cystoid macular edema, macular retinal pigment epithelium detachment, retinal pigment epithelium detachment, macular detachment, macular edema, retinal disorder, retinal exudates, retinal edema, retinal pigment epitheliopathy, retinopathy, and subretinal fluid.^18,32^ Moreover, version 4.0 of the National Cancer Institute Common Terminology Criteria for Adverse Events (NCI CTCAE) does not include a specific severity grading for serous retinopathy^18^ and there are limited data available to relate the severity of the event with either the likelihood of visual disturbance or longer-term visual impairment.^21^

There are many etiologies in clinical practice for the presence of subretinal fluid. In addition to clinical examination, ancillary imaging studies such as optical coherence tomography, fluorescein angiography, indocyanine green, and optical coherence tomography angiography are useful in determining the root pathophysiology of the retinopathy. Unlike more common etiologies of subretinal fluid, such as exudative choroidal neovascular membrane or central serous retinopathy, MEK inhibitor-associated subretinal fluid has a distinct fluorescein angiography pattern that does not display leakage. From this, it appears to be a distinct entity from the abovementioned retinopathies. From observational studies, it is known that these lesions fluctuate and resolve upon cessation of the medication. As a result, it is hypothesized that the mechanism of action of MEK inhibitor-associated retinopathy is through disruption of the retinal pigment epithelium resulting in impaired function and subsequent exudation.^20^ Given this clinical knowledge, management should begin with observation and should not immediately involve therapies such as anti-VEGF injections unless choroidal neovascularization is detected.

In conclusion, an overview of the available literature, clinical presentations, and key learnings from our clinical experience highlight best practices for managing MEK inhibitor-associated ocular toxicity. Patients should undergo regular ophthalmologic evaluations, beginning prior to initiation of MEK inhibitor therapy. For the majority of patients, dose interruption or reduction will not be required because the events are generally mild and self-limiting.

## Data Availability

No new data were generated or analyzed in support of this research.
